# Surface Engineering of Carbon-Based Microelectrodes for High-Performance Microsupercapacitors

**DOI:** 10.3390/mi10050307

**Published:** 2019-05-07

**Authors:** Liang He, Tianjiao Hong, Yue Huang, Biao Xiong, Xufeng Hong, Muhammad Tahir, Waqas Ali Haider, Yulai Han

**Affiliations:** 1 State Key Laboratory of Advanced Technology for Materials Synthesis and Processing, Wuhan University of Technology, Wuhan 430070, China; hongtj@whut.edu.cn (T.H.); hyhuangyuehy@gmail.com (Y.H.); nanobear@whut.edu.cn (B.X.); 1316824174@whut.edu.cn (X.H.); tahir@whut.edu.cn (M.T.); haider@whut.edu.cn (W.A.H.); 2 School of New Materials and New Energies, Shenzhen Technology University, Shenzhen 518118, China

**Keywords:** microelectrode, supercapacitor, carbon

## Abstract

In this research, the enhancement in electrochemical performance of pyrolyzed carbon microelectrodes by surface modification is investigated. For the proposed microfabrication process, pyrolyzed carbon microelectrodes with multi-walled carbon nanotubes (MWCNTs) on their surface are obtained by developing GM-1060 photoresist in mixture of propylene glycol methyl ether acetate (PGMEA) and CNTs, and following pyrolysis of a micropatterned photoresist. Polyvinyl alcohol (PVA)/H_2_SO_4_ electrolyte (1 M) was applied to assemble this carbon/CNT microelectrode-based all-solid-state microsupercapacitor (carbon/CNT-MSC). The carbon/CNT-MSC shows a higher electrochemical performance compared with that of pyrolyzed carbon microelectrode-based MSC (carbon-MSC). The specific areal and volumetric capacitances of carbon/CNT-MSC (4.80 mF/cm^2^ and 32.0 F/cm^3^) are higher than those of carbon-MSC (3.52 mF/cm^2^ and 23.4 F/cm^3^) at the scan rate of 10 mV/s. In addition, higher energy density and power density of carbon/CNT-MSC (2.85 mWh/cm^3^ and 1.98 W/cm^3^) than those of carbon-MSC (2.08 mWh/cm^3^ and 1.41 W/cm^3^) were also achieved. This facile surface modification and optimization are potentially promising, being highly compatible with modern microfabrication technologies and allowing integration of highly electrically conductive CNTs into pyrolyzed carbon to assemble MSCs with improved electrochemical performance. Moreover, this method can be potentially applied to other high-performance micro/nanostructures and microdevices/systems.

## 1. Introduction

Supercapacitors (SCs), an important kind of energy storage device, have many advantages over traditional energy storage devices [1,2,3]. There are three kinds of SCs employed in energy storage, namely electrical double layer capacitors (EDLCs), pseudocapacitors, and hybrid SCs. Among these three kinds of SCs, EDLCs are the most commonly applied and studied SCs due to their high stability and low cost. For EDLCs, the charge is stored electrostatically in the Helmholtz layer at the interface of the electrode and electrolyte. EDLC electrodes usually have high specific surface area (SSA) and electrical conductivity such as activated carbons (AC), carbon aerogels, carbon nanotubes (CNTs), graphene, carbide-derived carbon (CDC), and others [4]. Owing to their high SSA and that no chemical reactions occur between electrode and electrolyte, EDLCs usually possess high power density, fast charge/discharge rates, and ultralong lifetimes [5,6,7].

Microsupercapacitors (MSCs) with interdigital microelectrodes, showing relatively high SSA and short ion diffusion pathways, are of great potential for energy storage microdevices [8,9]. Various fabrication processes for MSCs have been proposed, including photolithography [10,11,12], inkjet printing [13], laser-irradiation assisted method [14,15,16], and others. Among these fabrication processes, photolithography is widely used due to its capability of facile control for interdigital microelectrode configuration and full compatibility with current microtechnologies [17,18,19]. Additionally, micropatterned photoresists can be directly carbonized into porous carbon microstructures which possess good chemical inertness, high reliability, and high electrical conductivity. Furthermore, the pyrolyzed carbon can be employed as a significant component in microdevices/systems such as microcantilevers [20], micromirrors [21], and MSCs [22,23,24,25,26,27,28]. In recent years, there are some reported results related to MSCs constructed by pyrolyzed carbon microelectrodes which aim to improve the electrical conductivity, energy storage performance, and cycling stability of pyrolyzed carbon microelectrodes. Yang et al. [26] fabricated an MSC based on photoresist/chitosan-coated carbon nanotube (CHIT-CNT) microelectrode, which has an excellent specific capacitance and high cycling stability over 10,000 cycles. The facile and compatible construction strategy is the critical issue for high-performance MSC.

The microfabrication process of MSC is more important than the choice of electrode materials regarding the reduction of costs. However, previous work has usually focused on how to fabricate the MSC more effectively and ignored the universality of the microfabrication process, and there are a lack of strategies for the optimization of the surface, especially for limited area of MSCs. Surface engineering is an effective way to further improve the performance of a supercapacitor, while rarely applied in MSCs. Herein, developing an advanced surface engineering method for MSCs will expand the optimization strategy to obtain higher electrochemical performance. In this research, a novel surface engineering approach for pyrolyzed carbon microelectrodes is reported. The carbon microelectrodes are derived from a micropatterned GM-1060 negative photoresist (Gersteltec Sarl), which is developed in a mixture of propylene glycol methyl ether acetate (PGMEA) developer and multi-walled carbon nanotubes (MWCNTs), followed by pyrolysis under N_2_ flow. By developing the photoresist in a mixture of PGMEA and CNTs, a micropatterned photoresist with CNTs on its surface is obtained. Subsequent pyrolysis of the micropatterned photoresist is conducted under N_2_ flow at 900 °C and 1 M polyvinyl alcohol (PVA)/H_2_SO_4_ electrolyte is applied as electrolyte to assemble the all-solid-state MSC. The specific areal capacitance of carbon-MSC is 3.52 mF/cm^2^ (23.4 F/cm^3^) at the scan rate of 10 mV/s, maximum energy density is 2.08 mWh/cm^3^ and maximum power density is 1.41 W/cm^3^. For carbon/CNT-MSC, a higher specific areal capacitance of 4.80 mF/cm^2^ (32.0 F/cm^3^) was obtained at the scan rate of 10 mV/s, maximum energy density was 2.85 mWh/cm^3^ and maximum power density was 1.98 W/cm^3^. By this facile surface modification method, a significant improvement in performance of MSC is achieved. Besides, this method could potentially be used for the application of high-performance microelectrodes.

## 2. Experimental

### 2.1. Preparation of the Developer

Fifty milligrams CNTs (purity > 99.9 wt %, XF NANO, Nanjing, China) and 20 mL PGMEA were uniformly mixed together and ultrasonicated for 1 h at room temperature. Afterwards, the mixture was used as the developer for GM-1060 photoresist.

### 2.2. Microfabrication process of Microsupercapacitor (MSC) with Carbon Nanotubes (CNTs) Modification

Firstly, Si/SiO_2_ substrate (3000/500 nm) was rinsed by acetone, isopropanol (IPA), and deionized (DI) water, followed by baking at 135 °C for 20 min. A uniform GM-1060 negative photoresist layer on the substrate was obtained by spin coating at 2000 rpm for 40 s. The sample was left to cool down to room temperature. After a 5 min, the sample was pre-baked at 65 °C for 5 min and then 95 °C for 30 min. Then the sample was exposed to UV light for photolithography. After 5 min, the sample was post-baked under the same conditions as in the pre-baking process, and was left to cool down to room temperature. Afterwards, the development and rinsing of samples were conducted in PGMEA/CNTs for 3 min and IPA for 30 s, respectively, followed by pyrolysis at 900 °C under N_2_ flow for 1 h.

### 2.3. Characterization and Electrochemical Performance Measurements

Morphology of the fabricated microelectrodes was studied using a field-emission scanning electron microscope (SEM, JEOL JSM-7100F, Tokyo, Japan) at an acceleration voltage of 15 kV. X-ray photoelectron spectroscopy (XPS) measurements were performed using a VG Multilab 2000 instrument for chemical bond characterization. Micro-Raman spectra of the samples were obtained by a Renishaw RM-1000 laser Raman microscope. The electrochemical performances of MSCs were evaluated by a commercial potentiostat (AC Instruments, 660D Model) in 1 M PVA/H_2_SO_4_ gel polymer electrolyte, which is a mixture of 5 mL H_2_SO_4_, 10 g PVA, and 100 mL deionized water. The potential window of the cyclic voltammetry tests was 0–0.8 V and the frequency range of electrochemical impedance spectroscopy (EIS) plots was 0.01–100,000 Hz.

## 3. Results and Discussion

**Figure 1:**
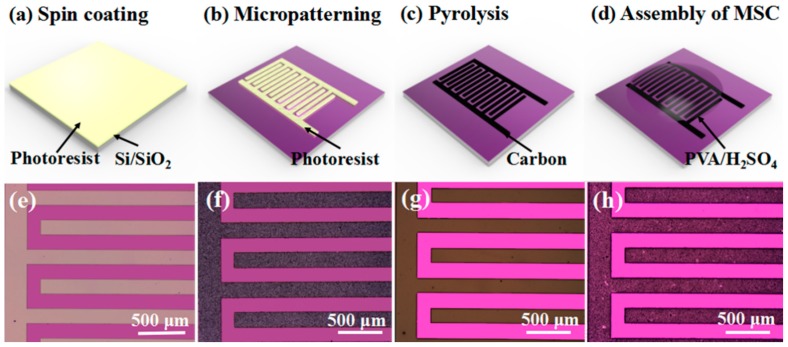
(**a**–**d**) Microfabrication process of carbon/CNT microelectrode-based all-solid-state microsupercapacitor (MSC) (carbon/CNT-MSC). (**e**) Optical image of micropatterned photoresist. (**f**) Optical image of micropatterned photoresist/CNTs. (**g**) Optical image of carbon microelectrodes. (**h**) Optical image of carbon/CNTs microelectrodes.

Figure 1a–d demonstrate the microfabrication process of MSC with CNT modification and include three processes: The Si/SiO_2_ substrate was cleaned, followed by a baking process. After that, spin coating and photolithography processes were conducted to obtain a uniform interdigital GM-1060 negative photoresist layer on the substrate. Afterwards, the sample was soaked in PGMEA/CNTs for development, followed by annealing treatment to obtain pyrolyzed carbon/CNT microelectrodes.

Figure 1e,g show optical images of the micropatterned photoresist and carbon microelectrodes, respectively. Figure 1f,h show the optical images of the micropatterned photoresist/CNTs and carbon/CNT microelectrodes, respectively. As shown in Figure 1e–h, it is obvious that the micropatterned photoresist and fabricated microelectrodes possess fine and complete micropatterns with no cracks, indicating that this surface modification method is highly suitable for the fabrication of microelectrodes. In addition, the color of the microelectrodes is much darker, and visual differences can be found due to CNT modification. By mixing CNTs with GM-1060 developer, CNTs can be attached to the surface of the photoresist, which will still have a stable attachment with carbon even after pyrolysis, due to the excellent thermal stability of CNTs.

**Figure 2:**
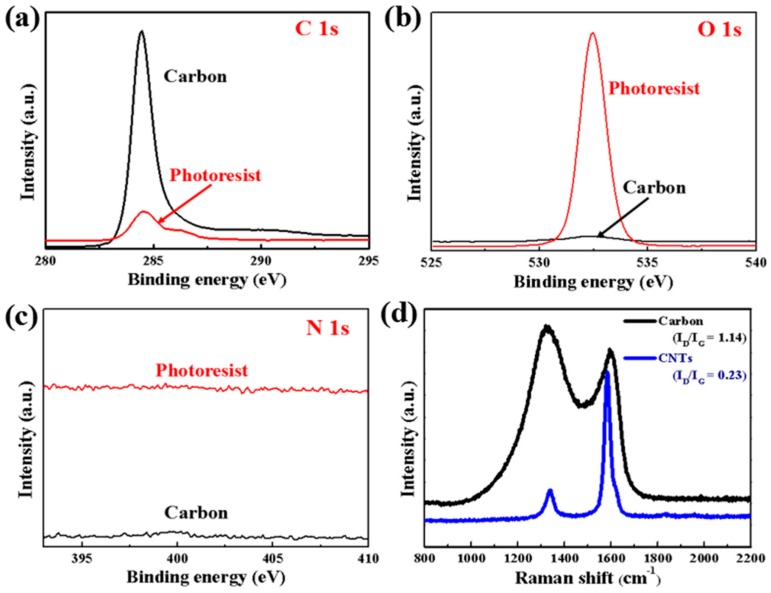
(**a**–**c**) XPS curves of GM-1060 photoresist and pyrolyzed carbon. (**d**) Raman spectra of pyrolyzed carbon and CNTs.

To investigate the state of related elements of GM-1060 before and after pyrolysis, X-ray photoelectron spectroscopy (XPS) was utilized. As shown in Figure 2a, there are main peaks at 284.6 eV for both GM-1060 photoresist and pyrolyzed carbon, indicating the presence of C–C in aromatic rings while a small shoulder at binding energy of ~286.1 eV for GM-1060 photoresist can be seen, implying contribution from different bonding configurations of carbon and oxygen. The intensity of the carbon peak of pyrolyzed carbon is higher than that of the photoresist. It is worth noting that after adsorbing CNTs onto the surface of GM-1060, a weaker C1s peak of that is obtained, due to the polymer coating. As described in Figure 2b,c, the oxygen peak and nitrogen peak of pyrolyzed carbon have disappeared (O1s peaks belong to the adsorbed oxygen in the air), which confirm that the composite of GM-1060 and CNTs has been pyrolyzed completely to carbon. The Raman spectra of pyrolyzed carbon and CNTs are described in Figure 2d to show their microstructure and graphitization degree. As shown in Figure 2d, both a graphitic band (G-band) and disorder-induced band (D-band) are observed. The peak at ~1350 cm^−1^ corresponds to the D-band of the microcrystallite graphite due to enhanced double resonance Raman scattering. Another peak at ~1590 cm^−1^ can be regarded as the result of a slight frequency shift of the single Raman line found at ~1575 cm^−1^ for single graphitic crystals that is ascribed to the bond stretching motion pairs of sp^2^ C atoms present in olefinic chains or aromatic rings [29]. The ratios (I_D_/I_G_) of CNTs (I_D_/I_G_ = 0.23) are much lower than that of pyrolyzed carbon (I_D_/I_G_ = 1.14), indicating the higher degree of graphitization and larger graphite crystallites in CNTs than in pyrolyzed carbon [30]. Herein, after compositing with CNTs, the average ratio (I_D_/I_G_) of carbon/CNTs is much higher than that of the pyrolyzed carbon. A higher degree of graphitization of pyrolyzed carbon means higher electrical conductivity, which would also be demonstrated in electrochemical performance.

**Figure 3:**
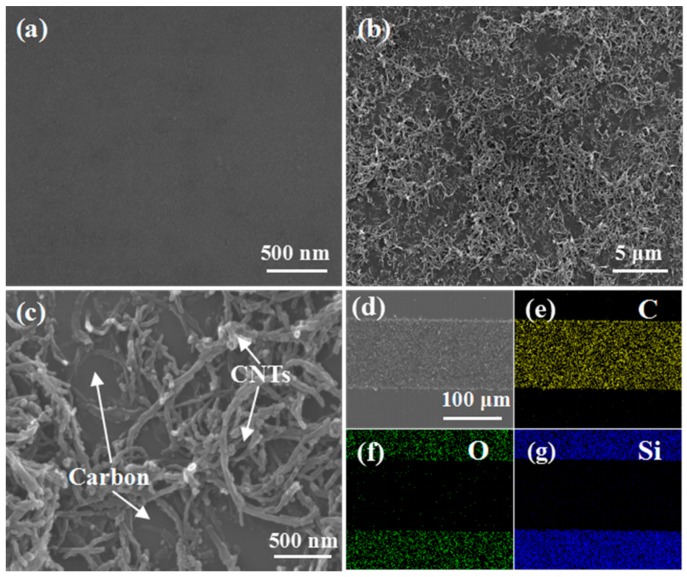
(**a**) SEM image of the surface of pyrolyzed carbon. (**b**) Low-magnification SEM image of carbon/CNT microelectrode surface. (**c**) High-magnification SEM image of carbon/CNT microelectrode surface. (**d**) SEM image of a carbon/CNT microelectrode. (**e**–**g**) Corresponding C/O/Si element mapping of carbon/CNTs microelectrode.

Figure 3a–c show SEM images of the surface of pyrolyzed carbon, and low-magnification and high-magnification SEM images of the carbon/CNT microelectrode surface. Different from the typical morphology of pyrolyzed carbon shown in Figure 3a, the CNT network is formed on the surface of carbon/CNT microelectrodes, as shown in Figure 3b,c. Interestingly, during the development process, the CNTs firmly adsorbed on the surface of GM-1060. After pyrolysis, CNTs near the surface of pyrolyzed carbon were well bonded with the surface of carbon. Figure 3d shows an SEM image of a carbon/CNT microelectrode. The networking structure of carbon/CNTs will provide higher surface area than that of pyrolyzed carbon for energy storage. Figure 3e–g show the C/O/Si element distribution in the microelectrodes, which indicates that C is the main element in pyrolyzed carbon.

**Figure 4:**
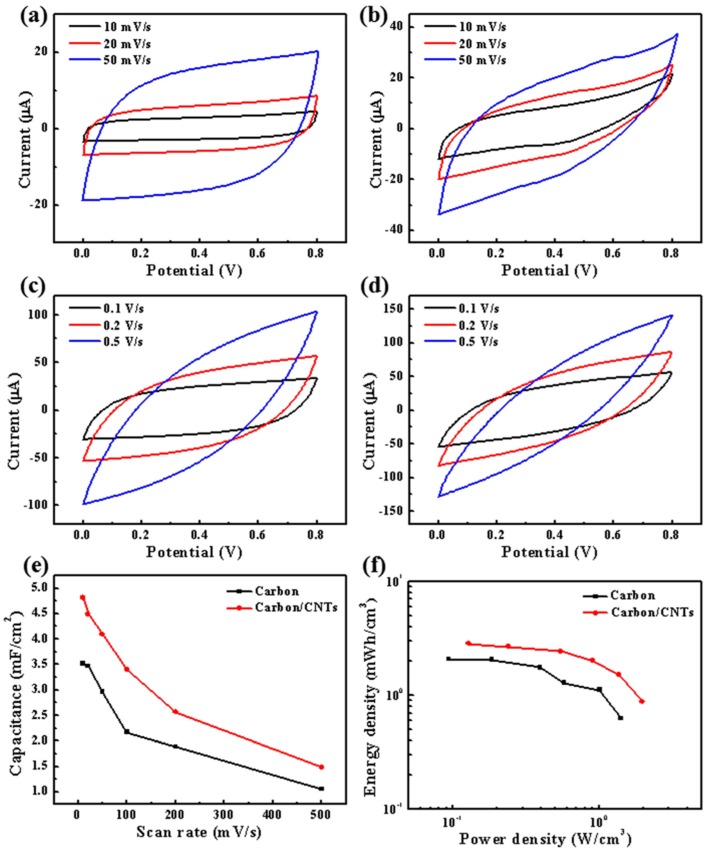
(**a**) CV curves of carbon-MSC at low scan rates. (**b**) CV curves of carbon/CNT-MSC at low scan rates. (**c**) CV curves of carbon-MSC at high scan rates. (**d**) CV curves of carbon/CNT-MSC at high scan rates. (**e**) Capacitance of carbon-MSC and carbon/CNT-MSC at different scan rates. (**f**) Energy density and power density of carbon-MSC and carbon/CNT-MSC, respectively.

Figure 4 shows the electrochemical performances of the carbon-MSC and carbon/CNT-MSC. CV curves of carbon-MSC with rectangular-like shapes at low scan rates are shown in Figure 4a, indicating the typical EDLC behavior of carbon-MSC. However, as shown in Figure 4b, the CV curves of carbon/CNT-MSC are different, which indicates that the electrochemical behavior has changed due to CNT modification on the microelectrode surface. With the increase of scan rate, as shown in Figure 4c,d, the shapes of CV curves gradually transform into a fusiform for both carbon-MSC and carbon/CNT-MSC, which could be due to the polarization. The areal capacitance (*C*_area_), stack capacitance (*C*_stack_), energy density (*E*), and power density (*P*) are calculated based on Equations (1)–(4) [31]:

(1) $$C_{\mathrm{area}}=\frac{\int I(V)dV}{2As\Delta V}$$

(2) $$C_{\mathrm{stack}}=\frac{C_{A}}{d}$$

(3) $$E=\frac{C_{\mathrm{area}}\Delta V^{2}}{7200d}$$

(4) $$P=\frac{E}{\Delta t}\times 3600$$

where ∫ *I*(*V*)*dV* is the integrated area of the CV curves and *A* is the area of the microelectrodes. *s* is the scan rate and Δ*V* is the potential window during CV tests. *d* is the thickness of microelectrodes and Δ*t* is the discharge time.

The specific areal capacitance calculated from CV curves of carbon-MSC is 3.52 mF/cm^2^ (23.4 F/cm^3^) at the scan rate of 10 mV/s. Its maximum energy density is 2.08 mWh/cm^3^ and maximum power density is 1.41 W/cm^3^. By contrast, the specific areal capacitance of carbon/CNT-MSC is 4.80 mF/cm^2^ (32.0 F/cm^3^) at the scan rate of 10 mV/s. In addition, its maximum energy density is 2.85 mWh/cm^3^, and maximum power density is 1.98 W/cm^3^. Based on the calculation, the relationship of capacitance of carbon-MSC and carbon/CNT-MSC at different scan rates, as well as energy density with power density of carbon-MSC and carbon/CNT-MSC, are presented in Figure 4e,f, respectively. It is obvious that the capacitance, energy density, and power density of carbon/CNT-MSC are higher than those of carbon-MSC. This result can be explained by the following two reasons: One is that the CNTs form a network structure on the surface of the microelectrodes which could increase the specific area of the microelectrodes, so that more ions can be absorbed and EDLC could be enhanced [32,33]. The other is that CNTs have higher electrical conductivity than pyrolyzed carbon, leading to faster charge transfer and higher response current during CV tests. Consequently, electrochemical performance can be improved by using this facile surface modification method for microelectrodes.

**Figure 5:**
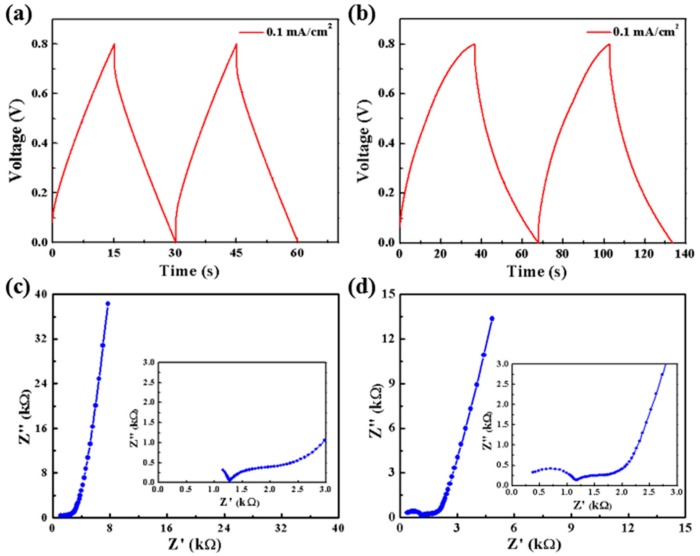
(**a**,**b**) GCD curves of carbon-MSC and carbon/CNT-MSC at a current density of 0.1 mA/cm^2^. (**c**,**d**) Electrochemical impedance spectroscopy (EIS) results of carbon-MSC and carbon/CNT-MSC with a frequency range from 0.01 to 100,000 Hz.

Galvanostatic charge-discharge (GCD) curves of carbon-MSC and carbon/CNT-MSC at a current density of 0.1 mA/cm^2^ are demonstrated in Figure 5a,b. It is obvious that it takes more than 60 s for carbon/CNT-MSC to conduct charge/discharge process, much longer than that of carbon-MSC [34]. Also, the IR drop in GCD curves decreases. These differences indicate the improvement in both electrical conductivity and capacitance, and correspond to the explanation of capacitance increment in Figure 4. Figure 5c,d show the EIS curves of carbon-MSC and carbon/CNT-MSC with the frequency ranging from 0.01 to 100,000 Hz. EIS results indicate that the inner resistance of the microelectrode decreased because CNTs have higher conductivity than pyrolyzed carbon and the networking structure contributes to the fast charge transfer. Table 1 clearly exhibits the performance comparison of this work and other carbon-based MSCs [35,36,37,38,39]. The fabricated all-solid-state MSC (carbon/CNT-MSC) shows excellent electrochemical performance for energy storage.

## 4. Conclusions

In this research, surface engineering of pyrolyzed carbon-based microelectrodes by microfabrication technology is proposed. It is found that the carbon/CNT-MSC shows higher electrochemical performance than the carbon-MSC. In addition, it is significant that our approach simplifies the microfabrication process of microelectrodes with surface modification by developing photoresist in a mixture of developer and CNTs. This facile surface modification method for microelectrodes is a highly promising approach to obtain high-performance microelectrodes, showing great potential in applications of carbon-based microelectrodes.

## Figures and Tables

**Table 1:** Performance comparison of carbon-based MSCs.

Fabrication Method	Specific Capacitance (mF/cm^2^)	Materials	Ref.
This work	4.80	Pyrolyzed carbon/CNTs	-
Mask-assisted filtration	9.8	Phosphorene/graphene	[35]
Lithography	5.9	Carbon	[36]
Preset filling	0.249	CNTs	[37]
Laser-assisted method	0.0627	Graphene	[38]
Nanoimprint lithography	0.008	Carbon	[39]
